# Expanding cholera serosurveillance to vaccinated populations

**DOI:** 10.1128/mbio.01898-25

**Published:** 2025-10-07

**Authors:** Forrest K. Jones, Taufiqur R. Bhuiyan, Damien M. Slater, Ralph Ternier, Kian Robert Hutt Vater, Ashraful I. Khan, Fahima Chowdhury, Kennia Visieres, Rajib Biswas, Mohammad Kamruzzaman, Edward T. Ryan, Stephen B. Calderwood, Regina C. LaRocque, Richelle C. Charles, Daniel T. Leung, Justin Lessler, Louise C. Ivers, Firdausi Qadri, Jason B. Harris, Andrew S. Azman

**Affiliations:** 1 Department of Epidemiology, Johns Hopkins Bloomberg School of Public Health 25802 , Baltimore, Maryland, USA; 2 Infectious Diseases Division, International Centre for Diarrhoeal Disease Research, Bangladesh (icddr,b) 56291, Dhaka, Bangladesh; 3 Division of Infectious Diseases, Massachusetts General Hospital 2348, Boston, Massachusetts, USA; 4 Zanmi Lasante, Port au Prince, Haiti; 5 Department of Biochemistry and Molecular Biology, Mawlana Bhashani Science and Technology University, Santosh 271385, Tangail, Bangladesh; 6 Department of Medicine, Harvard Medical School 205260 , Boston, Massachusetts, USA; 7 Department of Immunology and Infectious Diseases, Harvard T.H. Chan School of Public Health 1857 , Boston, Massachusetts, USA; 8 Division of Infectious Diseases, University of Utah School of Medicine 12348 , Salt Lake City, Utah, USA; 9 Division of Microbiology and Immunology, University of Utah School of Medicine 12348 , Salt Lake City, Utah, USA; 10 Department of Epidemiology, University of North Carolina Gillings School of Global Public Health 41474 , Chapel Hill, North Carolina, USA; 11 Carolina Population Center, The University of North Carolina at Chapel Hill 51765, Chapel Hill, North Carolina, USA; 12 Center for Global Health, Massachusetts General Hospital 536138, Boston, Massachusetts, USA; 13 Department of Global Health and Social Medicine, Harvard Medical School 1811 , Boston, Massachusetts, USA; 14 Harvard Global Health Institute 275971, Cambridge, Massachusetts, USA; 15 Department of Pediatrics, Harvard Medical School 548300 , Boston, Massachusetts, USA; 16 Centre for Emerging Viral Diseases, Geneva University Hospitals 27230 , Geneva, Switzerland; 17 Division of Tropical and Humanitarian Medicine, Geneva University Hospitals 27230 , Geneva, Switzerland; University of Pretoria, Pretoria, Gauteng, South Africa

**Keywords:** enteric bacteria, serology, immunoserology, epidemiology

## Abstract

Mass oral cholera vaccination campaigns targeted at subnational areas with high incidence are central to global cholera elimination efforts. Serological surveillance offers a complementary approach to address gaps in clinical surveillance in these regions. However, similar immune responses from vaccination and infection can lead to overestimates of the incidence of infection. To address this, we analyzed antibody dynamics in infected and vaccinated individuals to refine seroincidence estimation strategies for partially vaccinated populations. We tested 757 longitudinal serum samples from confirmed *Vibrio cholerae* O1 cases and uninfected contacts in Bangladesh as well as vaccinees from Bangladesh and Haiti, using a multiplex bead assay to measure IgG, IgM, and IgA binding to five cholera-specific antigens. Infection elicited stronger and broader antibody responses than vaccination, with rises in cholera toxin B-subunit (CTB) and toxin-coregulated pilus A (TcpA) antibodies uniquely associated with infection. Previously proposed random forest models frequently misclassified vaccinated individuals as recently infected (over 20% at some time points) during the first 4 months post-vaccination. To address this, we developed new random forest models incorporating vaccinee data, which kept false-positive rates among vaccinated (1%) and unvaccinated (6%) individuals low without a significant loss in sensitivity. Simulated serosurveys demonstrated that unbiased seroincidence estimates could be achieved within 21 days of vaccination campaigns by ascertaining the vaccination status of participants or applying updated models. These approaches to overcome biases in serological surveillance enable reliable seroincidence estimation even in areas with recent vaccination campaigns enhancing the utility of serological surveillance as an epidemiologic tool in moderate-to-high cholera incidence settings.

**IMPORTANCE:**

Serological surveillance can improve how we monitor cholera in high-burden areas where clinical surveillance is limited. However, vaccination can produce immune responses similar to infection, leading to overestimates in seroincidence. This study extends seroincidence estimation techniques using machine learning models to partially vaccinated populations. We analyzed antibody dynamics from vaccinated and infected individuals to develop methods that reduce the misclassification of vaccinated individuals as recently infected. These methods enable reliable seroincidence estimates in areas with recent vaccination campaigns, providing a step toward better epidemiologic monitoring in the context of global cholera control initiatives. Studies in other populations are needed to further validate our results and understand their generalizability.

## INTRODUCTION

Cholera remains a global public health threat, with an estimated 95,000 deaths per year (1). The Global Taskforce for Cholera Control’s End Cholera 2030 Roadmap is based on highly focused disease prevention and control in subnational “cholera hotspots” (2). Killed oral cholera vaccines (OCV) are one recommended component of cholera prevention in hotspots as they reduce severe cholera symptoms and limit transmission (3, 4). Clinical surveillance for *V. cholerae* infection is often limited in highly affected communities, with most infections being missed and many suspected cases of diarrheal illness being misattributed as cholera (5, 6). Serological surveillance (i.e. serosurveillance) has been proposed as a complementary approach to measure cholera transmission and burden (7). However, it is unclear whether current lab and analytic methods for serosurveillance are appropriate in vaccinated populations, where immune responses to vaccines could be misattributed to natural infections.

The two most commonly used bivalent OCVs in cholera-endemic regions (Shanchol and Euvichol) contain five inactivated strains of *V. cholerae* (both the O1 Inaba and Ogawa serotypes as well as the now extremely rare O139 serogroup) and no component of the cholera toxin (8). The vaccine is administered to individuals over the age of 1 year in two doses spaced at least 2 weeks apart, though one-dose use has become increasingly common due to the global shortage of vaccines (9). Mass campaigns are generally conducted in two rounds, with each round lasting only a few days to a week (10).

Similarities between the human antibody response to *V. cholerae* infection and OCV have been previously assessed (11). Vaccination boosts many of the same serological markers that have been proposed to identify previously infected individuals (7, 12). Therefore, seroincidence estimates from vaccinated populations could overestimate the true infection incidence by misclassifying vaccinated individuals as recently infected, potentially threatening the validity of this approach in cholera endemic areas globally.

We hypothesize that several strategies related to serosurvey design, laboratory protocols, and statistical analyses might mitigate the overestimation of seroincidence in vaccinated populations. Using new data on antibody kinetics following vaccination and *V. cholerae O1* infection, our study aims to quantify the extent of misclassification of vaccinated individuals as recently infected and provides a framework for adapting serosurveillance in populations that have benefited from cholera vaccination campaigns.

## RESULTS

We analyzed data from 236 samples collected from 43 Bangladeshi vaccine recipients (i.e., vaccinees) and 212 samples from 36 Haitian vaccine recipients, all of whom received two doses of the Shanchol vaccine, spaced 14 days apart (Table 1). Among the Bangladeshi participants, nearly half (47%) were less than 10 years old, and 49% were male. In contrast, all Haitian participants were adults (≥18 years old), with the majority (72%) being male.

**TABLE 1:** Individual characteristics of culture confirmed cholera patients and vaccinated individuals^*a*^

Characteristic	Bangladeshi cholera cases (*n* = 48)		Bangladeshi vaccinees (*n* = 43)		Haitian vaccinees (*n* = 36)	
	No.	%	No.	%	No.	%
Age group						
<5 years	8	17	7	16	0	0
5–9 years	14	29	14	33	0	0
10–17 years	9	19	4	9	0	0
≥18 years	17	35	18	42	36	100
Female	18	38	22	51	10	28
*V. cholerae* O1 Ogawa isolated	39	81	NA		NA	

^*a*^ Serological data were also available for three uninfected household contacts (41-year-old female , 11-year-old male, and 18-year-old male) of Bangladeshi cases enrolled with measurements taken at 2, 7, and 30 days after enrollment of the initial case. *V. cholerae* O1 Inaba was isolated from cases where Ogawa was not isolated. *V. cholerae* O1 isolation was not attempted in the cohorts of vaccinees. NA, not applicable.

Additionally, we included data from a Bangladeshi cohort of patients with *V. cholerae* infection, consisting of 300 samples from 48 individuals, primarily young (46% under 10 years old) and predominantly male (62%). Most of these individuals (81%) had *V. cholerae* O1 serotype Ogawa isolated from their stool, while the remainder were of the Inaba serotype. We also analyzed nine samples from three uninfected household contacts of patients with cholera (Table 1).

Baseline samples were collected from all vaccinated individuals on the day of their first dose. Bangladeshi vaccine recipients provided additional samples up to 42 days post-vaccination, while Haitian participants were followed for a longer duration, with samples collected up to 360 days post-vaccination (Table S1). Patients with *V. cholerae* O1 infection were followed for up to 1,080 days, with periodic blood sample collection (2–7 samples per individual). Household contacts each provided three serum samples within the first 30 days of follow-up.

### Immune response to vaccination and infection differs in magnitude and breadth

**Fig 1:**
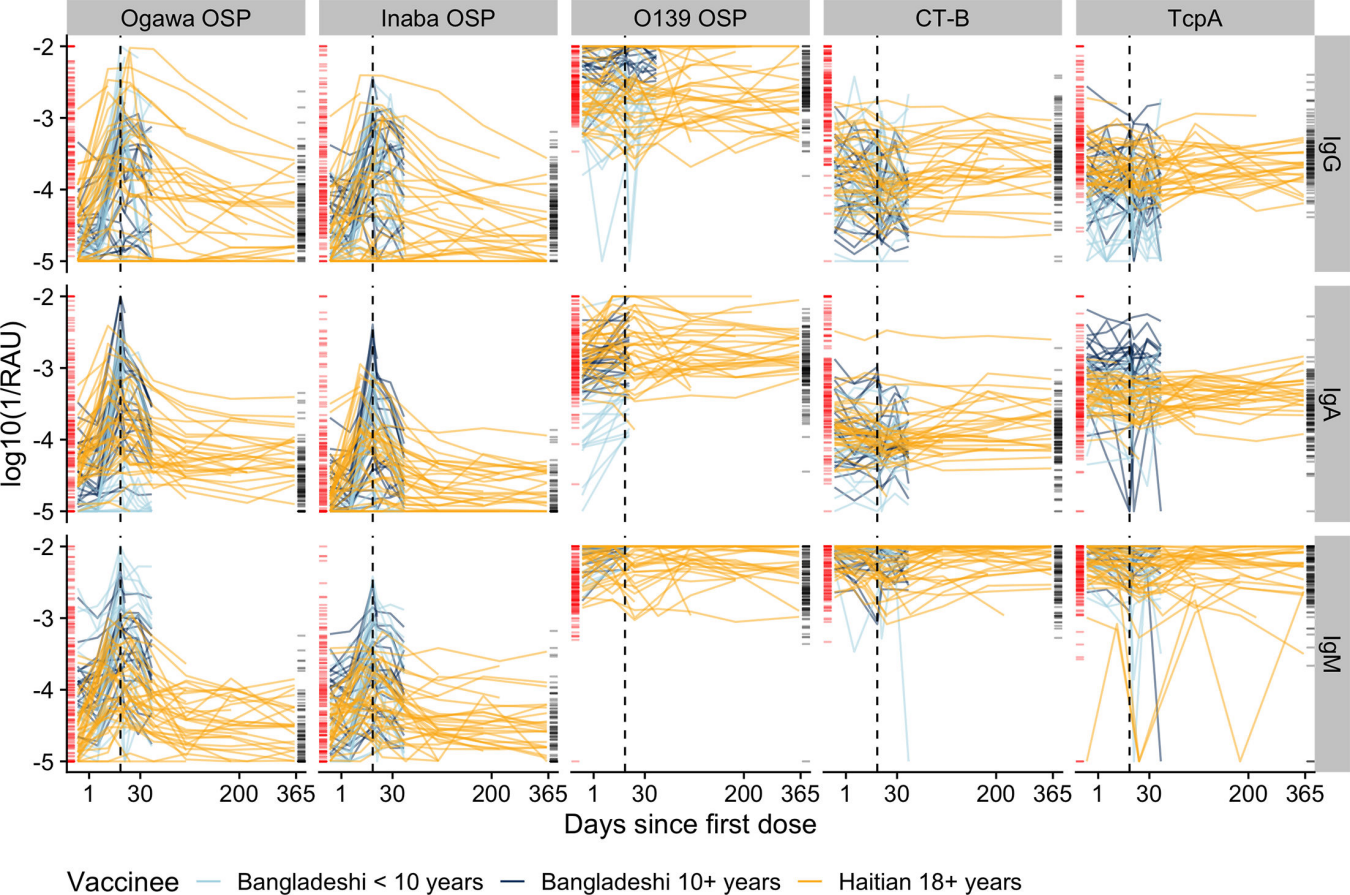
Multiplex bead assay measurements of IgG, IgM, and IgA against OSP, CTB, and TcpA antigens among vaccinated volunteers and comparison with cholera cases. The *Y*-axis indicates the concentration of antibodies shown as the log (base 10) of the inverse relative antibody unit (RAU). The *X*-axis indicates days since the first dose of vaccination, is square root-transformed. Each colored line indicates individual trajectories over time (light blue: Bangladeshi vaccinees <10 years, dark blue: Bangladeshi vaccinees ≥10 years, gold: Haitian vaccinees ≥18 years). Rug plots on the vertical axes show the antibody measurements from the cohort of cholera cases in Bangladesh (red: case measurements inside the 200-day infection window; black: uninfected household contacts and case measurements outside a 200-day infection window). The black dotted line indicates the timing of the second dose vaccination, 14 days after the first dose.

We identified differences in the types and quantity of antibodies among vaccinated and infected individuals. Bangladeshi patients with naturally occurring *V. cholerae* O1 infections and vaccinees had similar baseline IgG antibody distributions after stratifying by age group (<10 years vs 10+ years, Fig. 1; Fig. S1). Haitian vaccinees had lower baseline levels of anti-OSP IgG antibodies against both serotypes, Inaba and Ogawa. While both infection and vaccination led to large increases in anti-OSP antibodies against both serotypes, infection led to a more consistent and robust rise against both serotypes than vaccination (Fig. 1; Tables S2 and S3). IgG and IgA antibodies targeted at the cholera toxin (B subunit [CTB]) increased dramatically after infection but not vaccination, with neither exposure type leading to a rise in anti-CTB IgM. Despite the presence of *V. cholerae* O139 in the vaccine, only few vaccinees had more than a fourfold rise in anti-O139 OSP antibodies across isotypes (18% IgM, 22% IgG, and 42% IgA). Most cases (46% and 40%) but few vaccinees (14% and 3%) generated a greater than fourfold rise in anti-TcpA IgG and IgA (Table S3). When comparing samples collected at 180 days post-exposure to baseline, on average, IgG antibodies to CTB, Ogawa OSP, and Inaba OSP were more than twice as high in cases, while no antibody measurements in vaccinees were significantly different from baseline (Table S4). Lastly, we used multidimensional scaling to map the dynamic antibody profiles of those recently infected or vaccinated (200 days) and found that they form partially separated clusters (Fig. S2).

### Previous seroincidence models frequently misclassify vaccination as recent infection

We assessed how often vaccinated people were misclassified as seroincident (i.e., predicted to be recently infected based upon a serological response profile) using a previously published seroincidence model. This model, the infection-only model (Table 2), was trained on three IgG antibody measurements (Ogawa OSP, Inaba OSP, and CTB) from unvaccinated individuals in Bangladesh. We investigated four post-infection time periods (i.e., infection windows), where infections were considered recent: 45, 120, 200, and 300 days. These models were calibrated to classify no more than 5% of people not recently infected as seroincident (i.e., a nominal false positivity rate of 5%). For example, the 200-day model will only classify antibody profiles receiving at least 81% positive “votes” as seroincident.

**TABLE 2:** Seroincidence model descriptions

Name	Training data source	Markers	Classes
Infection-only model	Bangladeshi cholera patients^*a*^	Anti-CTB IgG Anti-Ogawa OSP IgG Anti-Inaba OSP IgG	Recently infected Not recently infected
Mixed-cohort two class model	Bangladeshi cholera patients^*a*^ Bangladeshi vaccinees Haitian vaccinees	Anti-CTB IgG Anti-Ogawa OSP IgG Anti-Inaba OSP IgG	Recently infected Not recently infected
Mixed-cohort three class model	Bangladeshi cholera patients^*a*^ Bangladeshi vaccinees Haitian vaccinees	Anti-CTB IgG Anti-Ogawa OSP IgG Anti-Inaba OSP IgG	Recently infected Recently vaccinated Neither

^*a*^ Serological data from three uninfected household contacts of Bangladeshi cases were also used.

**Fig 2:**
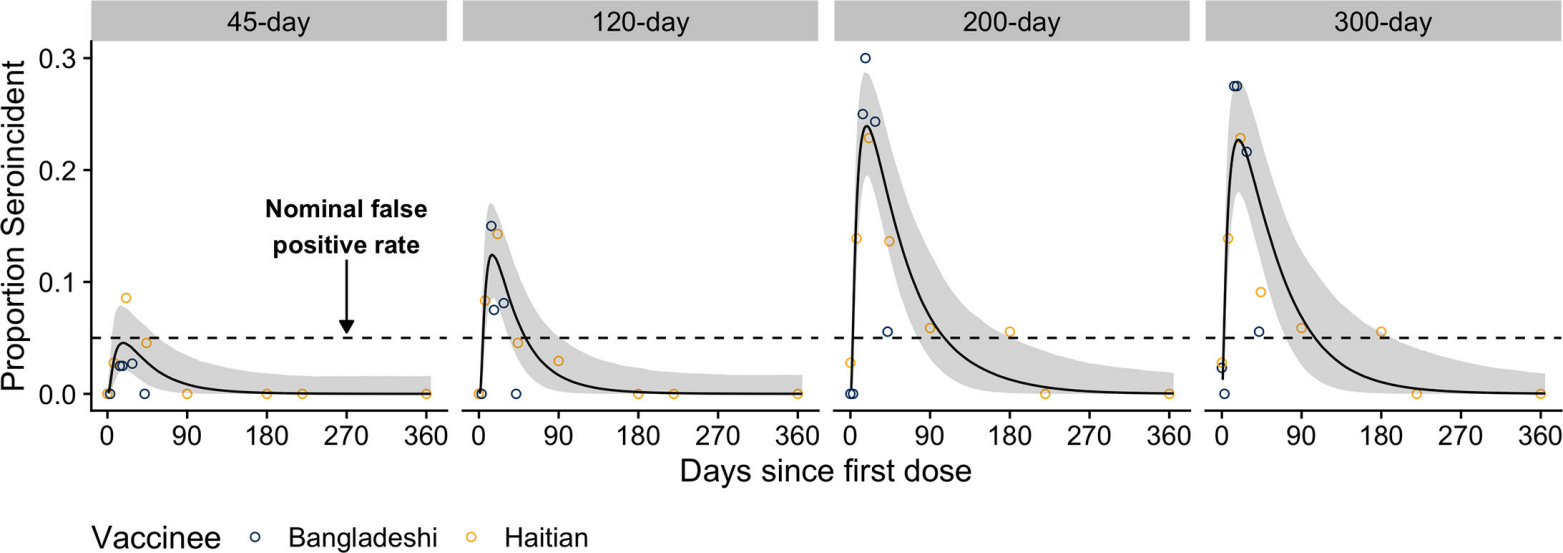
Misclassification of vaccinees as seroincident by previous seroincidence models using 45-, 120-, 200-, and 300-day infection windows. The model used (i.e., the infection-only model) to classify vaccinees as seroincident, or not, was a previously published random forest model trained on anti-CTB, anti-Ogawa OSP, and anti-Inaba OSP IgG measurements from Bangladeshi confirmed cases and uninfected household contacts. The proportion of Bangladeshi (dark blue) and Haitian vaccinees (gold) classified as seroincident is shown as dots. The overall proportion seroincident was modeled with a cubic function (black line and gray ribbon) using data from both cohorts of vaccinees. A black dashed line indicates the nominal false-positivity rate of 5%.

Models using a short infection window (e.g., 45 days) rarely misclassified vaccinees, though increasing the infection window led to longer periods of elevated misclassification (Fig. 2). With a 120-day infection window, this period lasted 47 days, peaking at 12% misclassification. The period of elevated misclassification grew longer (103 days and 102 days) with higher levels (24% and 23%) of misclassification when using models with 200- and 300-day infection windows. Misclassification did not differ substantially between the Haitian and Bangladeshi vaccinees (Fig. 2). Previously proposed models based on a larger set of serological markers and isotypes (IgA and IgM), including anti-TcpA and anti-O139 OSP antibodies, did not perform substantially better than those based on three IgG markers, and for shorter time windows, they led to greater misclassification (Fig. S3). For the 200-day window, the cross-validated area under the Receiver Operating Characteristic curve (AUC) did not differ significantly for the three IgG marker model (0.86) and the extended model (0.84).

### Inclusion of data from vaccinees improved the performance of seroincidence models without the need for additional antigen targets

**Fig 3:**
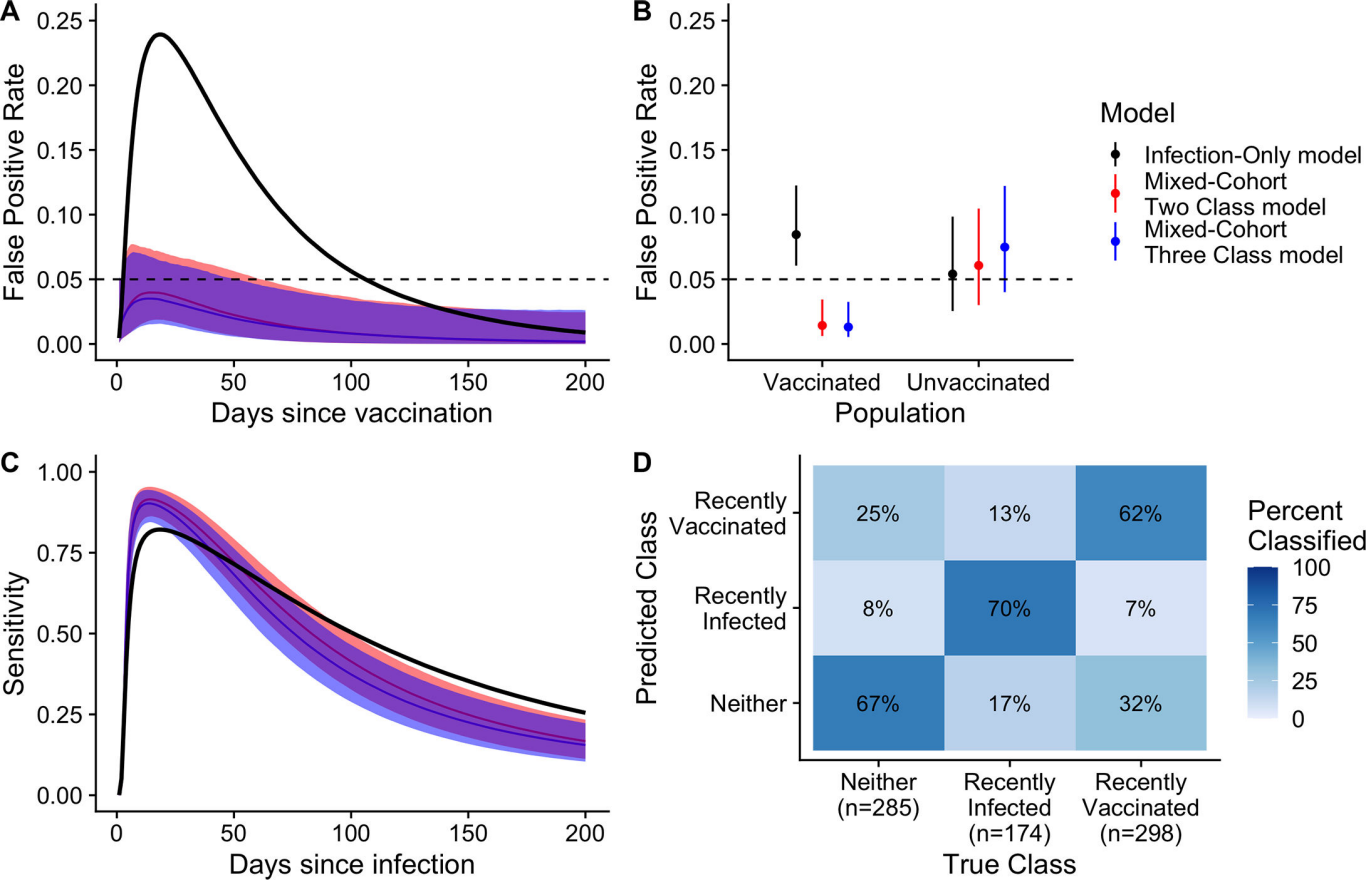
Comparison of the performance of random forest models when serological data from vaccinees are included in the training set. Individuals were considered recently infected or vaccinated if exposed in the last 200 days. (**A and B**) The black dashed line indicates the nominal false-positivity rate of 5%. (**A and C**) Solid lines show the median value, while shaded areas indicate the 95% credible interval. (**D**) Confusion matrix indicates the proportion of samples correctly classified from the new three-class model (mixed-cohort three-class model). Aside from the estimates for the false positivity rate among the vaccinated population for the infection-only model, all other parameters were estimated through leave-one-individual-out cross-validation.

We then evaluated the performance of two new random forest models (see Materials and Methods) to identify individuals infected in the last 200 days when trained with additional data from vaccinees. We chose to use the 200-day infection window as this was the shortest window where the misclassification began to increase (Fig. 2) and many, if not most, cholera outbreaks last less than 200 days (13). The mixed-cohort two class model was trained on cases, uninfected household contacts, and vaccinees and classified individuals as being recently infected or not recently infected (Table 2). To distinguish between recently infected, recently vaccinated (<200 days), and neither recently vaccinated/infected, we developed the mixed-cohort three class model, which we trained on the same data. Seroincidence models fit to serological data from both cases and vaccinees (Fig. 3A through C; Fig. S4) were less likely to misclassify vaccinees as seroincident while maintaining sensitivity to detect recent infections.

For both mixed-cohort models, the estimated false positivity rate among vaccinees was consistently below 5% between 0 and 200 days post-vaccination (Fig. 3A). Among vaccinees, the estimated false positivity rate (averaged over the 200-day period after vaccination) was much higher for the infection-only model (8%) than both mixed-cohort models (1%; Fig. 3B). Among unvaccinated individuals, the estimated false-positivity rate was only slightly lower for the infection-only model (5%) compared to the mixed-cohort models (6% and 7%) (Fig. 3B). All three models had similar average sensitivity to detect individuals infected in the last 200 days (range: 44%–51%) and similar patterns of time-varying sensitivity, peaking within the first 30 days after infection (Fig. 3C). The addition of anti-O139 OSP and anti-TcpA antibody measurements, as well as IgA and IgM measurements of all antibodies, resulted in only marginal improvements in performance (Fig. S5) and increased the cross-validated AUC from only 91% to 93%.

In some situations, it may be useful to not only distinguish between recently infected and not recently infected people but also to identify recently vaccinated people from their serologic signatures. We investigated the performance of the mixed-cohort three class model to further distinguish recently vaccinated (<200 days) individuals from those without a recent infection. The model correctly classified most samples, including 70% (121/174) from recently infected, 61% (184/298) from recently vaccinated, and 67% (192/285) from individuals neither infected nor vaccinated recently (Fig. 3D). Samples from both recently infected individuals (30/174, 17%) and recently vaccinated individuals (94/298, 32%) were more often misclassified as neither.

### Multiple adjustment strategies address bias in seroincidence estimates in partially vaccinated populations

We simulated serological surveys conducted after vaccination campaigns to assess the extent of bias in seroincidence estimates attributable to vaccination (i.e., the percent increase in estimated seroincidence relative to the true incidence of infection, which was set at 10 infections per 100 individuals in the last 200 days) (Methods S1). We investigated this bias when serosurveys were conducted 21 days (i.e., when the infection-only model has a high false-positive rate) and 120 days (i.e., sufficient time for the false-positive rate of the infection-only model to drop to its nominal rate) after a vaccination campaign.

**Fig 4:**
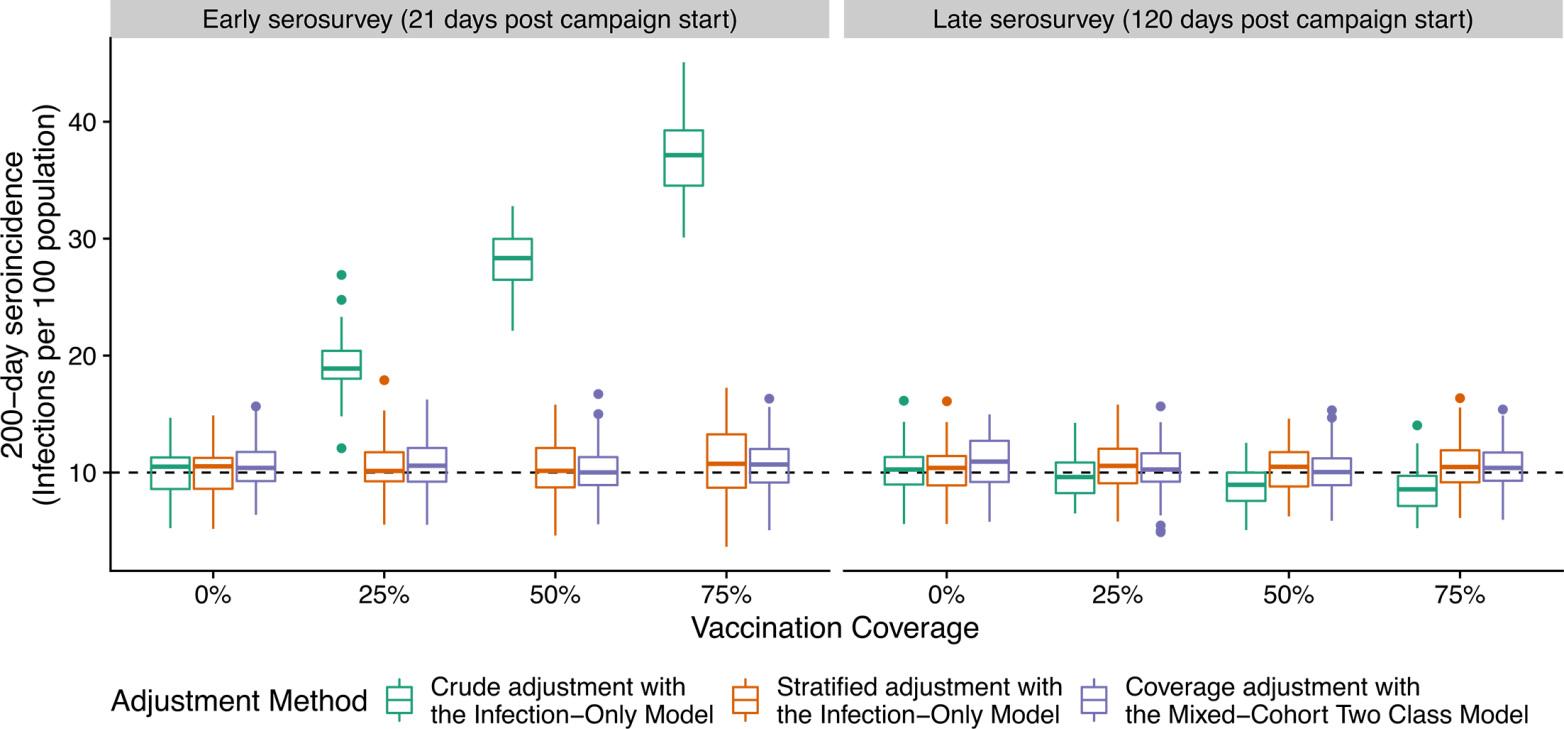
Simulated serological surveys in partially vaccinated settings using multiple strategies to calculate seroincidence 21 days (early) and 120 days (late) post-vaccination campaign. Boxplots show the distribution of 100 estimates of seroincidence using different strategies at various levels of vaccine coverage (*x*-axis). Each simulated survey had 1,000 persons. The true incidence was 10 infections per 100 in 200 days, as indicated by the dashed line.

As an example, a serosurvey of 1,000 participants might have 102 participants truly infected in the last 200 days and 527 vaccinated participants. Due to imperfect sensitivity and false positives from the infection-only model, 187 participants might be classified as seroincident (21 infected only, 105 vaccinated only, 32 both, and 29 neither) if the serosurvey was done 21 days after a vaccination campaign. By adjusting the serosurvey results using estimates of sensitivity (51%) and the false-positivity rate (5%) from using the infection-only model (without accounting for increased misclassification among vaccine recipients), the magnitude of bias in seroincidence estimates varied with both the timing of the survey and the vaccination coverage. When the “true” 200-day incidence of infection was set at 10 infections per 100 individuals, serosurveys conducted 21 days post-campaign produced estimates with higher bias as vaccination coverage increased. Specifically, the mean bias was 94% (range: 26%–159%) at 25% vaccination coverage and approximately 273% (range: 198%–420%) at 75% coverage (Fig. 4). However, if the survey had been conducted 120 days after the campaign instead, only 96 of the participants in the example might be classified as seroincident (21 infected only, 14 vaccinated only, 32 both, and 29 neither). For serosurveys conducted 120 days after the vaccination campaign, estimates based on the infection-only model consistently showed no discernible bias after adjusting for sensitivity and the false positivity rate (Fig. 4).

We found that two strategies (Table 3; Methods S1) mitigated bias in seroincidence estimates when surveys were conducted shortly after the vaccination campaigns. First, we continued using seroincident classifications from the infection-only model but modified the adjustment to account for the vaccine-status-specific performance. In this scenario, despite an elevated seroincidence among the vaccinated population, we adjust using a higher false-positivity rate (24%) for vaccinated individuals. At 50% vaccination coverage, adjusting for misclassification based on the individual-vaccination status produced an average estimate of 10% (range: 5%–16%) (Fig. 4).

**TABLE 3:** Description of strategies for serosurveys to address misclassification due to vaccination campaigns

Name	Data collected (sample size)	Prediction model used	Statistical adjustment of predictions
Crude adjustment with the infection-only model	Serological survey (*n* = 1,000)	Infection-only model	Incidence rate is adjusted by sensitivity and false-positivity rate to estimate the overall seroincidence
Stratified adjustment with the infection-only model	Serological survey and questionnaire data (*n* = 1,000)	Infection-only model	Vaccination status specific incidence rates are adjusted by their respective sensitivity and false-positivity rate Vaccination status specific seroincidence rates are combined to estimate the overall seroincidence
Coverage adjustment with the mixed-cohort two class model	Serological data (*n* = 1,000) Vaccine coverage data (*n* = 500)	Mixed-cohort two class model	Coverage data inform the expected false-positivity rate in the population Incidence rate is adjusted by sensitivity and false-positivity rate to estimate the overall seroincidence

As an alternative strategy, we used the mixed-cohort two class model with adjustment for model performance where the expected false-positive rate is informed by a vaccination coverage estimate. Returning to the example with 102 participants infected in the last 200 days and 527 vaccinated participants 21 days prior, now only 87 participants might be classified as seroincident by the mixed-cohort two class model. In this scenario, the participant vaccination status is unknown (e.g., samples taken from a blood bank), but coverage might be estimated to be 56% from a separate rapid coverage survey. We could then adjust by the sensitivity (46%) and an overall false positivity rate (5%) (calculated by weighing estimates of false positivity from among vaccinated and unvaccinated by the coverage estimate). At 50% vaccination coverage, using the mixed-cohort two class model with an accurate estimate of vaccine coverage yielded an average estimate of 10% (range: 5%–17%) (Fig. 4).

## DISCUSSION

Despite similarities in the immune response between infection and vaccination, our work illustrates that estimating cholera seroincidence through serological surveillance is feasible in partially vaccinated populations. We found that vaccination stimulates only a subset of the antibody repertoire of that generated by infection, and when a marker was stimulated after vaccination, the observed increase was often less than half as much as observed after infection. Due to these different post-exposure kinetics, excess misclassification of recently vaccinated individuals as seroincident only occurs when samples are collected within 4 months of vaccination. Accurate estimation of seroincidence can be made with a new generation of models proposed in this paper, simply by waiting a few months before conducting a serosurvey, or through collecting and incorporating data on vaccination coverage, at the individual or population level.

Vaccination and infection lead to measurable differences in the antibody response, which can be harnessed for serological surveillance. Though anti-Ogawa OSP and anti-Inaba OSP antibodies increased after both infection and vaccination, the smaller increase in magnitude differentiates vaccinees from cases. Anti-CTB and anti-TcpA IgG and IgA antibodies were only stimulated by infection, and their presence could be used to distinguish infection from vaccination, though misclassification with this approach could occur with children, who may have higher levels of anti-CTB antibodies (due to enterotoxigenic *Escherichia coli* [ETEC] infection) and are generally less likely to generate anti-TcpA antibodies than adults (14). Lastly, we found that few anti-O139 OSP antibodies were generated from vaccination (similar to previous findings [15]) and are unlikely to effectively differentiate vaccinees from infected individuals. Therefore, we do not expect vaccination with a monovalent version of OCV without an O139 strain, which was WHO-prequalified in mid-2024, to be any more challenging to differentiate from infection than current bivalent OCV (16).

Stimulation of anti-OSP antibodies through vaccination sometimes led to overestimation of seroincidence, but we proposed multiple ways to address this in the context of cross-sectional serological surveys. First, we found that models trained to identify individuals infected in the last 120 days sometimes misclassified vaccinated individuals as recently infected, though this slightly elevated misclassification occurred only briefly (i.e. within 47 days). As the duration of most cholera outbreaks has been documented to be less than 120 days (13), estimating the incidence in a cross-sectional survey shortly after an outbreak ends may have minimal bias using standard seroincidence models, especially if the vaccination campaign occurs early on in the outbreak. For situations where an infection window of 200 days is being considered, we found that misclassification can likely be addressed either through using new seroincidence models where vaccinated individuals have been used in the training sets and/or through ascertaining the individual-level vaccination status (e.g., through a questionnaire). However, the adjustment we proposed assumes that all individuals are vaccinated within a short window of time and requires estimates of sensitivity and specificity for the classification models. When starting a cross-sectional serosurvey, models to identify recent infections would ideally be validated with longitudinal serum samples from cases and vaccinees from a similar setting and target population.

This study comes with some notable limitations. The differences in the serological data among cases and vaccinees in this study may be partially attributed to differences in the age distribution and history of cholera transmission in the area shortly before sample collection. Also, our study population only contained individuals who were infected with *V. cholerae* O1 or received two doses of OCV, though none with both a known recent infection and vaccination. As cholera vaccination becomes more common, future serological studies will allow the capture of post-infection kinetics of individuals with a history of vaccination. Challenge studies, where exposure can be directly controlled, might also be well-suited for gathering serological data on individuals with different combinations of exposures. ETEC infection, which is prevalent in many locations with the incidence of cholera, stimulates cross-reactive antibodies that can bind to CTB (17) and may also cause excess misclassification by seroincidence models. As we did not have samples from a cohort of ETEC cases, we could not investigate this beyond observing high correlation of markers for antibodies binding to LT-B and CTB in children and adults in each cohort. We also did not investigate the dynamics after only single-dose vaccination, though previous work indicates limited additional immunogenicity from the second dose, though decay rates could differ (18, 19). Though vaccination campaigns often strive to provide two doses for each participant, it is common that participants receive only one dose, possibly impacting serosurveillance efforts. Lastly, though our simulation results indicate there are reasonable strategies to address excess misclassification due to vaccination, these simulations rely on several assumptions including that individuals both vaccinated and infected would have the same antibody dynamics as someone who was not vaccinated but infected.

Estimating the incidence of infection and/or disease is essential for identifying priority areas for cholera prevention, control measures, and the allocation of scarce resources like vaccines. Seroincidence estimation using cross-sectional serosurveys offers a valuable method to assess infection incidence, especially in settings where clinical surveillance data may be unreliable. Our study highlights several promising strategies to mitigate the overestimation of cholera seroincidence in partially vaccinated populations. While additional data across diverse populations are needed to evaluate the generalizability of our findings, these results suggest that the proposed methods are feasible and practical in the increasingly vaccinated landscape of cholera-affected regions.

## MATERIALS AND METHODS

### Study population

In this study, we analyzed data from three cohorts: confirmed cholera cases and their household contacts enrolled in Bangladesh as well as volunteers vaccinated with Shanchol enrolled in Bangladesh and Haiti (i.e., vaccinees).

In Bangladesh, as described previously, consenting patients aged more than 1 year hospitalized at the International Center for Diarrheal Disease Research, Bangladesh (icddr,b) Dhaka hospital with culture-confirmed *V. cholerae* O1 were enrolled between 2006 and 2018 (20, 21). We utilized data from a previous analysis where we selected a subsample of 51 confirmed cases (2–1,080 days post symptom onset) and uninfected contacts enrolled in Bangladesh (309 serum samples in total) (12). We ensured representation from both child and adult cases and used previously generated serological data to choose participants predictive of the remaining cohort.

In Bangladesh, as described previously, a non-inferiority trial comparing two killed, whole-cell cholera vaccines (Shanchol and Cholvax, Incepta [a locally produced OCV in Bangladesh]) was conducted in Mirpur, Dhaka, Bangladesh, between April 2016 and April 2017 (22). This study included 2,052 healthy individuals from 1 to 45 years old, who were given two doses of the vaccine at 14 days apart from one another. We selected a convenience sample of vaccinees who received Shanchol, including both children and adults, to test for this study. To characterize immunogenicity due to vaccination, serum samples were collected up to 42 days after the first dose of vaccination, where participants visited the field clinic up to six times to provide blood samples.

In Haiti, serum samples were collected from healthy volunteers ≥ 18 years old agreeing to receive the Shanchol vaccine enrolled from the outpatient department at Saint Nicholas Hospital in St. Marc, Haiti, an urban center in the Artibonite Department (23). Individuals were excluded if previously given OCV, pregnant, or reported to have had active gastrointestinal disorder within 7 days prior to enrollment. All individuals received two doses of Shanchol spaced 14 days apart. Vaccinees were enrolled in 2015 (May) and 2016 (January, March, and April). Samples were collected around 0, 7, 21, 44, 90, 180, 270, and 365 days after the first dose. While cholera cases were reported regularly in Haiti after being introduced in 2010, cases steadily decreased from 2011 through the time of these studies (24). We had access to samples of 73 vaccinees followed up to 360 days. After limiting selection to 67 individuals (92%) with at least three samples, we randomly selected 36 vaccinees.

### Multiplex bead serological testing and data processing

Based on a review of the published literature on immune responses to *V. cholerae* infection (12, 25–27) and our prior work (12), we focused our analysis on five cholera-related antigens using a multiplex bead assay, chosen based on their potential responsiveness to either infection or vaccination. These included O1 serogroup Ogawa serotype O-specific polysaccharide (OSP-BSA, part of the LPS), O1 serogroup Inaba serotype OSP, cholera toxin B-subunit (CTB), toxin co-regulated pilus subunit A (TcpA), and O139 OSP (*V. cholerae* O139 serogroup is no longer a cause of considerable human *V. cholerae* infections yet is included in the most commonly used bivalent OCVs). While not analyzed in this study, plates also included beads for cholera toxin holotoxin (CTH), *V. cholerae* cytolysin (VCC) (also known as hemolysin A), *V. cholerae* sialidase, heat-labile enterotoxin subunit B (LTB), heat-labile holo-enterotoxin (LTH) (expressed during ETEC infection), and influenza hemagglutinin 1 (as a control antigen). All antigens were conjugated to Luminex magnetic beads, as previously described (12).

All plates included a dilution series (from pooled convalescent sera of culture-confirmed *V. cholerae* O1 cases in Bangladesh) and control wells, all of which were run in triplicate. Following the testing protocol, serum, beads, and secondary antibodies binding to IgG, IgA, and IgM were added to each well. Given the limited amount of O139 serogroup OSP available, we were only able to test 167/236 (29%) Bangladeshi vaccinee samples for O139 OSP IgA and IgM; all samples from the cohorts were tested for these markers. Samples were run on a Luminex Flexmap 3D machine at Massachusetts General Hospital (28). Bead counts and median fluorescence intensity (MFI) values were exported from the xPONENT software program (version 4.3.1). Plates were retested when over half of the positive control dilutions had ≥5 antigens (excluding O139 OSP as they were not on all plates) with a coefficient of variation (calculated from triplicate MFI measurements) greater than 20%.

For the analysis, any measurements with a bead count of less than 30 were excluded (<0.5% of data points) to obtain statistically robust and representative MFI values, consistent with industry standards. MFI values were averaged across replicate wells. As previously described (12), we standardized MFI values from the assay to help adjust for plate-to-plate variability by calculating the relative antibody unit (RAU), the expected dilution of the pooled convalescent sera that would yield an equivalent MFI on this plate (29). Specifically, for each plate, we fit a log-logistic model, a flexible class of model used frequently to model dilution series. We used the median parameter estimates from the model to predict the RAU for each sample on the plate (30, 31). For samples with a predicted RAU outside the range of 10^5^ and 10^2^, the RAU was set at the threshold value.

The complete laboratory protocol for the MBA assay is available at https://doi.org/10.17504/protocols.io.3byl4b1x8vo5/v1.

### Statistical analyses

We compared the antibody kinetics of vaccinated volunteers and confirmed cholera cases by describing the average baseline, the geometric mean RAU ratio of the peak versus baseline of antibody measurements, and the proportion of individuals with an RAU ratio greater than fourfold. We used multidimensional scaling (32, 33) to visualize the immunologic profiles (in two dimensions) of samples from individuals infected or vaccinated within the last 200 days, including all markers previously mentioned.

Using the ranger package (34), we trained random forest classification models to determine if a sample was from a recently infected individual based on antibody measurements. We define samples classified as recently infected as seroincident. We explored several different periods for defining a recent infection (<45 days, <120 days, <200 days, and <300 days) corresponding to when samples from the Bangladeshi case data were collected. The models relied on measurements of three IgG markers (anti-Ogawa OSP, anti-Inaba OSP, and anti-CTB) shown to rise after infection, which current seroincidence models depend on (12). In supplemental analyses, we also explored model performance where we included IgG, IgM, and IgA markers (anti-Ogawa OSP, anti-Inaba OSP, anti-CTB, and anti-TcpA, respectively) as well as anti-O139 OSP IgG measurements in the models.

Each random forest model contained 1,000 classification trees, each of which “votes” for a sample as recently infected or not. Instead of choosing the class (i.e., seroincident or not) identified by the majority of votes, we established cut-offs for a nominal 5% false-positivity rate among unvaccinated individuals using cross-validation. Specifically, we randomly selected 50% of the samples, trained a random forest model to predict serostatus, and selected the lowest cutoff (e.g., proportion of votes that need to predict positive to classify a result as positive) that led to no more than 5% of unvaccinated true-negative individuals in the held-out set to be misclassified as seroincident. For each model, we repeated this cross-validation procedure 100 times and obtained the median value of the cut-off values to be used. While a 5% false positivity rate threshold was used for model calibration, this rate represents a balance between sensitivity and specificity and may require adjustment for other surveillance settings.

We fit three different types of random forest models: infection-only model, mixed-cohort two class model, and mixed-cohort three class model (Table 2). The infection-only model (used in Jones et al. [12]) is based on training data only from cholera cases and their household contacts in Bangladesh. The mixed-cohort two class model is also trained on these data as well as vaccinee data, which are not considered recently infected. The mixed-cohort three class model is also trained on case, household contact, and vaccinee data, but samples are classified as recently infected (<200 days), recently vaccinated (<200 days), or neither. When the mixed-cohort three class model classifies a sample, it simply selects the class with the highest number of votes among the three classes.

We assessed the accuracy of random forest models using leave-one-individual-out cross-validation (LOOCV) to calculate the time-varying false-positivity rate among vaccinees, time-varying sensitivity among cases, and the false-positivity rate among unvaccinated individuals. For time-varying false positivity and time-varying sensitivity models, we assumed that the logit (proportion seroincident) was a cubic function of (natural log-transformed) days since infection with a random intercept for each individual. For the false-positivity rate among unvaccinated individuals, we assumed that the logit (positivity) was only a random-intercept (i.e., per person) model. We report 95% credible intervals (CI).

To evaluate strategies for mitigating bias in seroincidence estimates, we simulated serosurveys to estimate 200-day seroincidence at both 21 days (early) and 120 days (late) after vaccination campaigns (Table 3). We evaluated three strategies: crude adjustment with the infection-only model (i.e., no adjustment taking into account the vaccination status), stratified adjustment with the infection-only model (i.e., adjust estimates for differential model performance by the vaccination status of the individual)*,* and coverage adjustment with mixed-cohort two class model (i.e., predict who is recently infected with the mixed-cohort two class model). We ran 100 simulations per scenario.

In each simulated serosurvey, 1,000 participants were randomly selected from an area with a constant infection incidence of 10% in the previous 200 days (Methods S1). Vaccination coverage was set at variable levels (0%, 25%, 50%, and 75%). Once vaccination status and days since last infection were randomly drawn for each person, we simulated their seroincident status for the random forest model used. Individuals who were both recently infected and vaccinated were assumed to have the immunological profile of those who were recently infected but not vaccinated. To account for individual-level variability in the immune response, each serosurvey participant was randomly assigned the identification number of one of the participants from the study cohort, conditional on their simulated recent infection status and vaccination status. Then, using the modeled estimates of individual probabilities of being classified seroincident (described above), we simulated their seroincident status. We then calculated the estimates of population-level seroincidence after adjusting for overall test misclassification (Methods S1).

## Data Availability

Data and code used to conduct statistical analyses are available at https://github.com/HopkinsIDD/cholera-vaccine-serosurvey.

## SUPPLEMENTAL MATERIAL

Supplemental material mbio.01898-25-s0001.pdf Supplemental text, tables, and figures.

ASM does not own the copyrights to Supplemental Material that may be linked to, or accessed through, an article. The authors have granted ASM a non-exclusive, world-wide license to publish the Supplemental Material files. Please contact the corresponding author directly for reuse.
