# Supplementary material for: Expanding cholera serosurveillance to vaccinated populations
Source: mBio. 2025 Oct 7;16(11):e01898-25. doi: 10.1128/mbio.01898-25 (PMC12607766; doi:10.1128/mbio.01898-25)

Supplementary Material

This file contains supplementary tables and figures for

“Expanding cholera serosurveillance to vaccinated populations” by Jones et al.

Methods S1. Description of the simulation of serosurveys and adjustment for misclassification

Simulation of serosurveys

For each simulated serosurvey, individual-level infection and vaccination status were generated using random draws. Each survey consisted of 1,000 individuals, with infection status and vaccination status sampled independently for each individual *i*. True infection status R_i was drawn from a Bernoulli distribution with probability equal to the specified seroincidence ($R_i \sim$ Bernoulli(λ)), and vaccination status V_i was drawn from a Bernoulli distribution with probability equal to the vaccination coverage ($V_i \sim$ Bernoulli(γ)). For recently infected individuals (R_i=1), the time since infection ("days ago") was sampled uniformly between 0 and 200 days. This process was repeated independently for each simulation replicate, producing a list of simulated datasets.

Conditional on these true statuses, seropositivity for each individual was generated using the predicted probability of being classified as seropositive from random forest models trained on cohort data of previously observed cholera cases and vaccinated individuals. These probabilities came from the models used to analyze cross-validation the results from the random forest models. Specifically, we already fit models to calculate the time-varying false positivity rate among vaccinees, time-varying sensitivity among cases, and the false positivity rate among unvaccinated individuals. For time-varying false positivity and time-varying sensitivity models, we assumed that the logit (proportion seroincident) was a cubic function of (natural log-transformed) days since infection with a random-intercept for each individual. For the false positivity rate among unvaccinated individuals, we assumed that the logit (positivity) was only a random-intercept (i.e., per person) model. In Figure 3, the overall estimates for these parameters are shown.

To account for individual-level variability in the immune response, each serosurvey participant was randomly assigned the identification number one of the participants from a study cohort, conditional on their simulated recent infection status and vaccination status. Then, using the modeled estimates of individual probabilities of being classified seroincident, we simulated their seroincident status (denoted as S_i for the Infection-Only Model or Z_i for the Mixed-Cohort Two Class Model) using a Bernoulli distribution. For infected individuals, seropositivity was determined independent of vaccination status. For uninfected individuals, seropositivity was determined separately for vaccinated (V_i=1) and unvaccinated (V_i=0) individuals using the corresponding model-predicted probability for each individual. These individual-level probabilities were only used to generate the simulated seropositive outcomes; all adjustment strategies described below use population-level sensitivity and false positivity parameters rather than the individual-level probabilities.

These simulated datasets were then analyzed using three adjustment strategies. In all likelihood expressions below, T=t denotes the timing of the vaccination campaign (i.e., the number of days since the campaign) relative to the serosurvey, which affects the expected false positivity due to vaccination.

Adjustments to estimate incidence of infection

Crude adjustment with the Infection-Only Model

Seroincident statuses (S) of participants were generated from estimates from the *Infection-Only Model*, denoted S_i for each individual i. For this crude adjustment method, vaccination status is ignored, so all individuals are treated as unvaccinated (V_i assumed 0) regardless of their true simulated vaccination status. The likelihood of observing the predicted seroincident statuses of all individuals in the serosurvey under this assumption is:

$$Pr(S \mid \lambda, T = t) = \prod [\, Pr(S_i \mid V_i = 0, R_i = 0)\,(1 - \lambda) + Pr(S_i \mid V_i = 0, R_i = 1)\,\lambda \,]$$

where the conditional probabilities $Pr(S_i \mid V_i{=}0, R_i{=}r)$ reflect the population-level sensitivity and false positivity rate for unvaccinated individuals.

Stratified adjustment with the Infection-Only Model

For this adjustment method, both the seroincident statuses (S) and the vaccination statuses (V) of participants are incorporated into the analysis. For real-world application, a questionnaire could be administered to participants to infer vaccination status at the time of sample collection. The likelihood for the observed data accounts for both misclassification and the population-level probability of vaccination γ (i.e., vaccine coverage):

$$Pr(S, V \mid \lambda, \gamma, T = t) = \prod [\, Pr(S_i \mid V_i, R_i = 0, T = t)\, Pr(V_i \mid \gamma)\,(1 - \lambda) +$$

$$Pr(S_i \mid V_i, R_i = 1, T = t)\, Pr(V_i \mid \gamma)\,\lambda \,]$$

where $Pr(V_i{=}1 \mid \gamma) = \gamma$ and $Pr(V_i{=}0 \mid \gamma) = 1{-}\gamma$, and $Pr(S_i \mid V_i, R_i{=}r, T{=}t)$ reflects the population-level sensitivity and false positivity rate for each vaccination-infection stratum.

Coverage adjustment with the Mixed-Cohort Two Class Model

Seroincident statuses were generated from estimates from the *Mixed-Cohort Two Class Model*, denoted Z_i, to distinguish it from S_i. A rapid coverage survey of 500 individuals was incorporated to inform the expected false positivity rate in the population. Let X denote the number of vaccinated individuals in the rapid coverage survey, where X ~ Binomial(500, γ). The proportion vaccinated is then estimated as X/500. For uninfected individuals, the probability of being classified as seroincident by this model is:

$$Pr(Z_i = 1 \mid R_i = 0, T = t) = Pr(Z_i = 1 \mid V_i = 0, R_i = 0)\,(1 - X/500) +$$

$$Pr(Z_i = 1 \mid V_i = 1, R_i = 0)\,(X/500)$$

The full likelihood for the observed seroincident statuses from the Mixed-Cohort Two Class model (Z) is:

$$Pr(Z \mid \lambda, T = t) = \prod [\, Pr(Z_i \mid R_i = 0, T = t)\,(1 - \lambda) + Pr(Z_i \mid R_i = 1, T = t)\,\lambda \,]$$

where the conditional probabilities $\Pr(Z_i \mid R_i=r)$ reflect the population-level sensitivity and false positivity rate.

Table S1. Timing of sample collection for each cohort

Exposure Type	Cohort	Days since exposure	Individuals	Samples (%)
Vaccination with Shanchol	Bangladeshi volunteer	0	43	43 (100)
Vaccination with Shanchol	Bangladeshi volunteer	3	43	40 (93)
Vaccination with Shanchol	Bangladeshi volunteer	14	43	40 (93)
Vaccination with Shanchol	Bangladeshi volunteer	17	43	40 (93)
Vaccination with Shanchol	Bangladeshi volunteer	28	43	37 (86)
Vaccination with Shanchol	Bangladeshi volunteer	42	43	36 (84)
Vaccination with Shanchol	Haitian volunteer	0	36	36 (100)
Vaccination with Shanchol	Haitian volunteer	7	36	36 (100)
Vaccination with Shanchol	Haitian volunteer	21	36	35 (97)
Vaccination with Shanchol	Haitian volunteer	44	36	22 (61)
Vaccination with Shanchol	Haitian volunteer	90	36	34 (94)
Vaccination with Shanchol	Haitian volunteer	180	36	18 (50)
Vaccination with Shanchol	Haitian volunteer	220	36	8 (22)
Vaccination with Shanchol	Haitian volunteer	360	36	23 (64)
Natural infection with *V. cholerae* O1	Bangladeshi case-patient	2	48	48 (100)
Natural infection with *V. cholerae* O1	Bangladeshi case-patient	7	48	46 (96)
Natural infection with *V. cholerae* O1	Bangladeshi case-patient	30	48	46 (96)

Exposure Type	Cohort	Days since exposure	Individuals	Samples (%)
Natural infection with *V. cholerae* O1	Bangladeshi case-patient	90	48	42 (88)
Natural infection with *V. cholerae* O1	Bangladeshi case-patient	180	48	40 (83)
Natural infection with *V. cholerae* O1	Bangladeshi case-patient	270	48	12 (25)
Natural infection with *V. cholerae* O1	Bangladeshi case-patient	360	48	14 (29)
Natural infection with *V. cholerae* O1	Bangladeshi case-patient	540	48	25 (52)
Natural infection with *V. cholerae* O1	Bangladeshi case-patient	720	48	1 (2)
Natural infection with *V. cholerae* O1	Bangladeshi case-patient	900	48	25 (52)
Natural infection with *V. cholerae* O1	Bangladeshi case-patient	1080	48	1 (2)
Household member of case-patient infected with *V. cholerae* O1	Bangladeshi volunteer	2	3	3 (100)
Household member of case-patient infected with *V. cholerae* O1	Bangladeshi volunteer	7	3	3 (100)
Household member of case-patient infected with *V. cholerae* O1	Bangladeshi volunteer	30	3	3 (100)

Table S2. Geometric mean fold-rise in relative antibody unit by marker among cases and vaccines

For each individual, the fold-rise of RAU was calculated by comparing baseline and peak values**.** A t-test was used to test the statistical significance of the difference between cases and vaccinees.

Marker		Geometric mean fold-rise of RAU		
**Antigen**	**Isotype**	**Case**	**Vaccinee**	**p-value**
CTB	IgA	23.3	1.2	<0.01
CTB	IgG	23.9	1.5	<0.01
CTB	IgM	1.5	1.1	<0.01
Inaba OSP	IgA	15.1	6.6	0.01
Inaba OSP	IgG	12.5	6.1	0.01
Inaba OSP	IgM	13.2	4.4	<0.01
O139 OSP	IgA	2.1	2	0.69
O139 OSP	IgG	1.7	1.6	0.64
O139 OSP	IgM	1.5	1.4	0.28
Ogawa OSP	IgA	24.5	8.2	<0.01
Ogawa OSP	IgG	33.4	9.7	<0.01
Ogawa OSP	IgM	44.9	6.7	<0.01
TcpA	IgA	3.4	1.3	<0.01
TcpA	IgG	4.1	1.7	<0.01
TcpA	IgM	1.8	1.2	<0.01

Table S3. Proportion of individuals with a four fold-rise in RAU by marker among cases and vaccines
For each individual, the fold-rise of RAU was calculated by comparing baseline and peak values. Fischer's exact test was used to test the statistical significance of the difference between cases and vaccinees.

Marker		Proportion of individuals with >4-fold rise of RAU		
**Antigen**	**Isotype**	**Case**	**Vaccinee**	**p-value**
CT-B	IgA	96%	1%	<0.01
CT-B	IgG	98%	11%	<0.01
CT-B	IgM	8%	0%	0.02
Inaba OSP	IgA	73%	54%	0.04
Inaba OSP	IgG	73%	51%	0.02
Inaba OSP	IgM	71%	46%	<0.01
O139 OSP	IgA	15%	17%	0.81
O139 OSP	IgG	10%	8%	0.75
O139 OSP	IgM	4%	6%	1
Ogawa OSP	IgA	83%	59%	<0.01
Ogawa OSP	IgG	85%	61%	<0.01
Ogawa OSP	IgM	90%	57%	<0.01
TcpA	IgA	40%	3%	<0.01
TcpA	IgG	46%	14%	<0.01
TcpA	IgM	10%	1%	0.03

Table S4. Geometric mean ratio of measurements taken at the 180 day visit relative to baseline measurements for Bangladeshi cases and Haitian vaccinees

For each individual, the fold-rise of RAU was calculated by comparing baseline and measurements taken at 180 days. Bangladeshi vaccinees were not included as they did not have measurements collected past 45 days. The p-value (listed in parentheses) corresponds to the t-test of the logarithm (base 10) of the ratio relative to the null value (i.e. zero).

**Marker**		**Geometric mean fold-rise of RAU at 180 days (p-value)**	
**Antigen**	**Isotype**	**Case**	**Vaccinee**
CTB	IgA	1.7 (<0.01)	1 (0.78)
CTB	IgG	3.2 (<0.01)	0.9 (0.56)
CTB	IgM	1 (0.9)	0.9 (0.21)
Inaba OSP	IgA	1.4 (<0.01)	1.2 (0.17)
Inaba OSP	IgG	3 (<0.01)	1.2 (0.41)
Inaba OSP	IgM	1.2 (0.05)	0.9 (0.64)
O139 OSP	IgA	0.9 (0.42)	0.9 (0.31)
O139 OSP	IgG	1 (0.83)	0.9 (0.36)
O139 OSP	IgM	1.1 (0.39)	0.9 (0.32)
Ogawa OSP	IgA	1.8 (<0.01)	1.1 (0.51)
Ogawa OSP	IgG	8.4 (<0.01)	1.3 (0.19)
Ogawa OSP	IgM	1.8 (<0.01)	1.2 (0.5)
TcpA	IgA	1.2 (0.14)	0.8 (0.03)
TcpA	IgG	1.7 (<0.01)	0.8 (0.04)
TcpA	IgM	1.1 (0.41)	0.6 (0.18)

Figure S1. Comparison of baseline measurements of IgG markers by cohort and age group

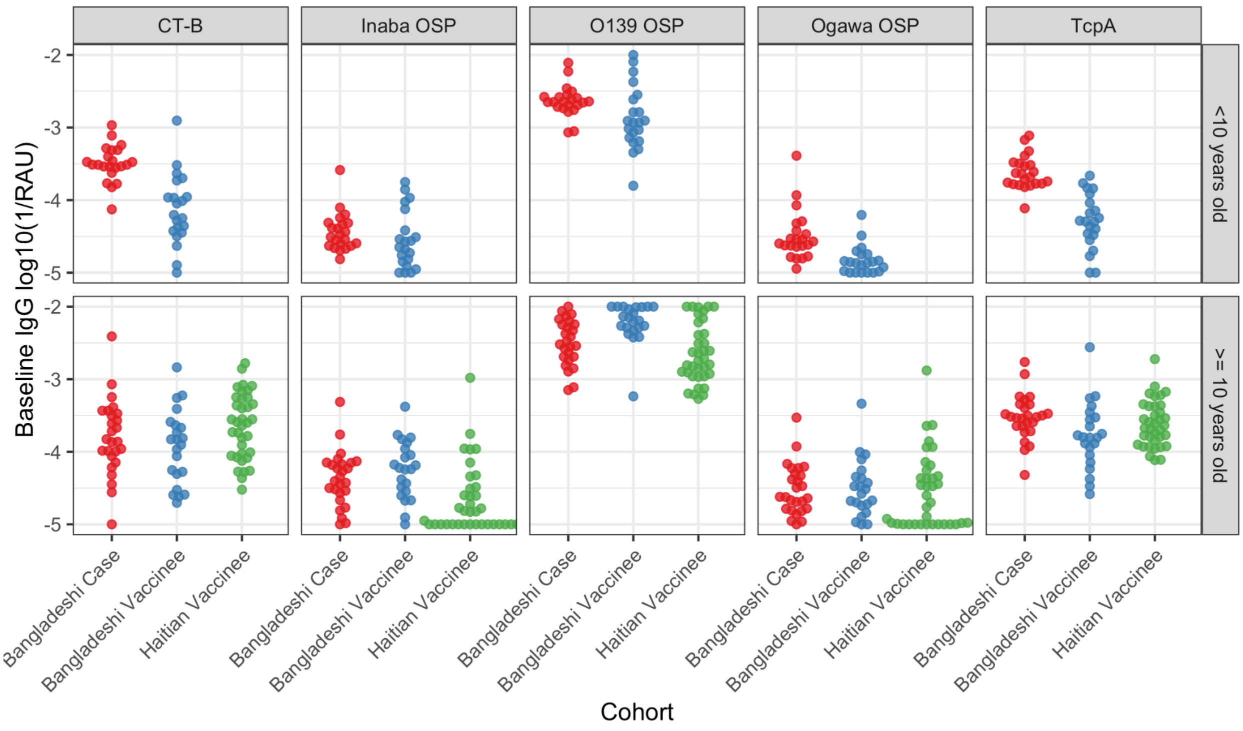

Figure S2: Multidimensional scaling analysis of all serological data collected

Each panel includes the two dimensions calculated from data collected within the specified time window. Black points represent the location of the centroids for the two new dimensions of cases (circle) and vaccinees (triangle).

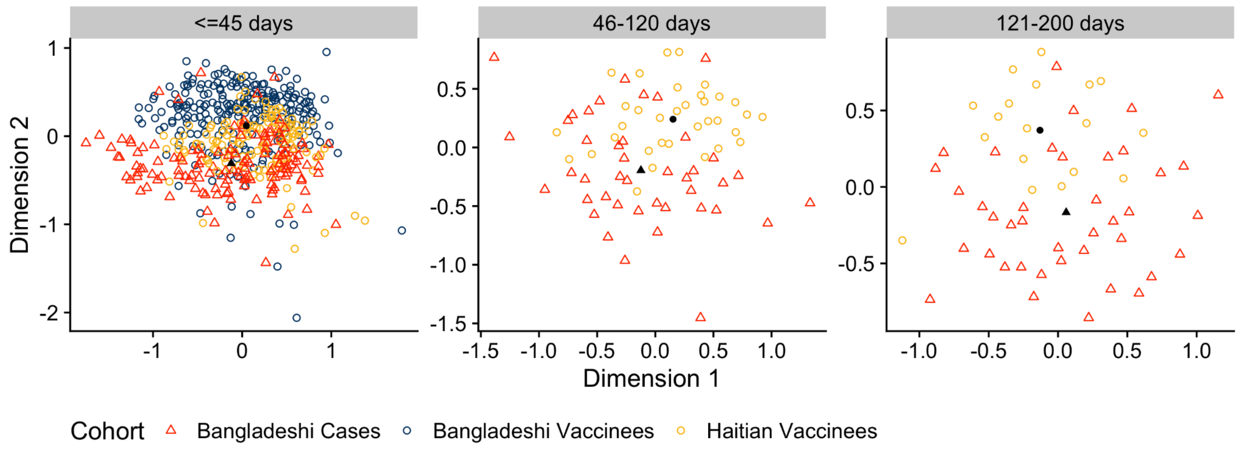

Figure S3: Misclassification of vaccinees as seroincident by previous seroincidence models with additional markers using 45, 120, 200, and 300 day infection windows. The model used (i.e., the Infection-Only Model) to classify vaccinees as seroincident or not was a previously published random forest model trained on anti-CTB, anti-Ogawa OSP, anti-Inaba OSP, anti-TcpA antibodies (IgG, IgM, and IgA) as well as anti-O139 OSP IgG from Bangladeshi confirmed cases and uninfected household contacts. The proportion of Bangladeshi (dark blue) and Haitian vaccinees (gold) classified as seroincident are shown as dots. The overall proportion seroincident was modeled with a cubic spline (black line and grey ribbon) using data from both cohorts of vaccinees. Black dashed line indicates the nominal false positivity rate of 5%.

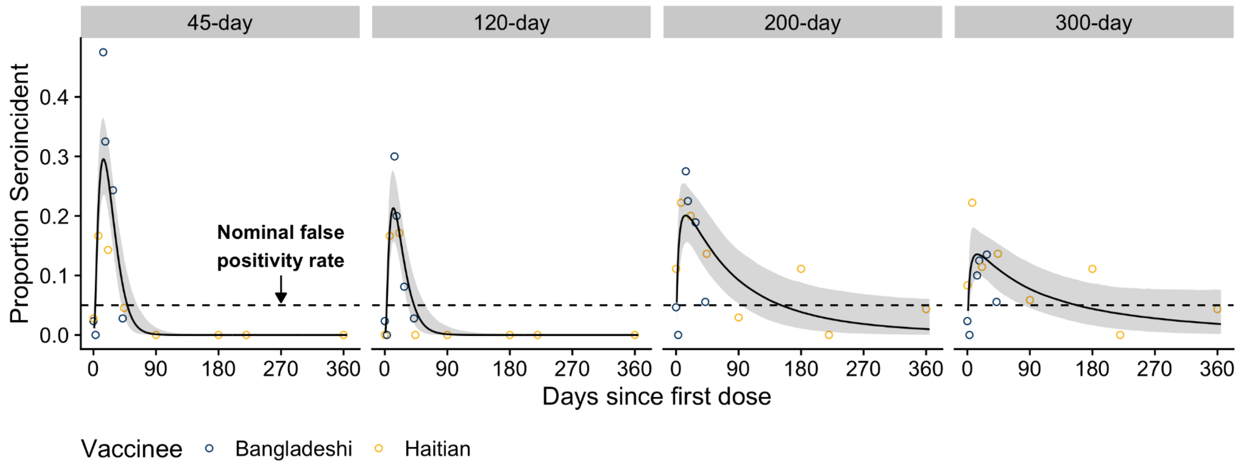

Figure S4: Confusion matrices for 200-day Infection-Only Model and Mixed-Cohort Two Class Model, using three IgG markers and all markers. The number and percent predicted for each true status is shown in each box, with blue shading corresponding to the percent. Aside from the predictions for the vaccinee data for the *Infection-only Model*, all other parameters were estimated through leave-one-individual-out cross-validation.

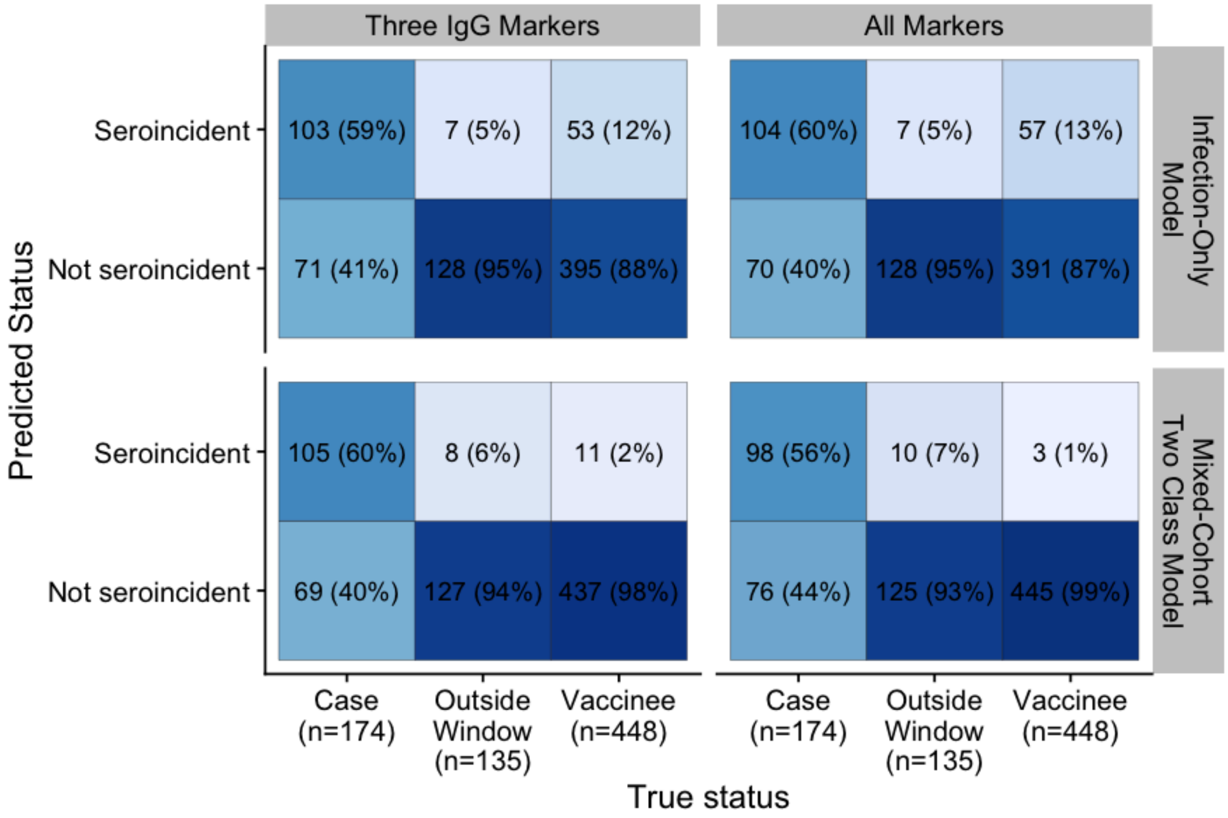

Figure S5: Comparison of performance of random forest models when all IgG, IgM, and IgA serological data from vaccinees are included in the training set. These random forest models used measurements of anti-CTB, anti-Ogawa OSP, anti-Inaba OSP, anti-TcpA antibodies (IgG, IgM, and IgA) as well as anti-O139 OSP IgG. Individuals were considered recently infected or vaccinated if exposed in the last 200 days. (A & B) Grey dashed line indicates the expected/nominal false positivity rate of 5%. (A & C) Solid lines show the median value while shaded areas indicate the 95% credible interval. (D) Confusion matrix indicates the proportion of samples correctly classified from the new three-class model (*Mixed-Cohort Three Class Model*). Aside from the estimates for the false positivity rate among the vaccinated population for the *Infection-only Model*, all other parameters were estimated through leave-one-individual-out cross-validation.

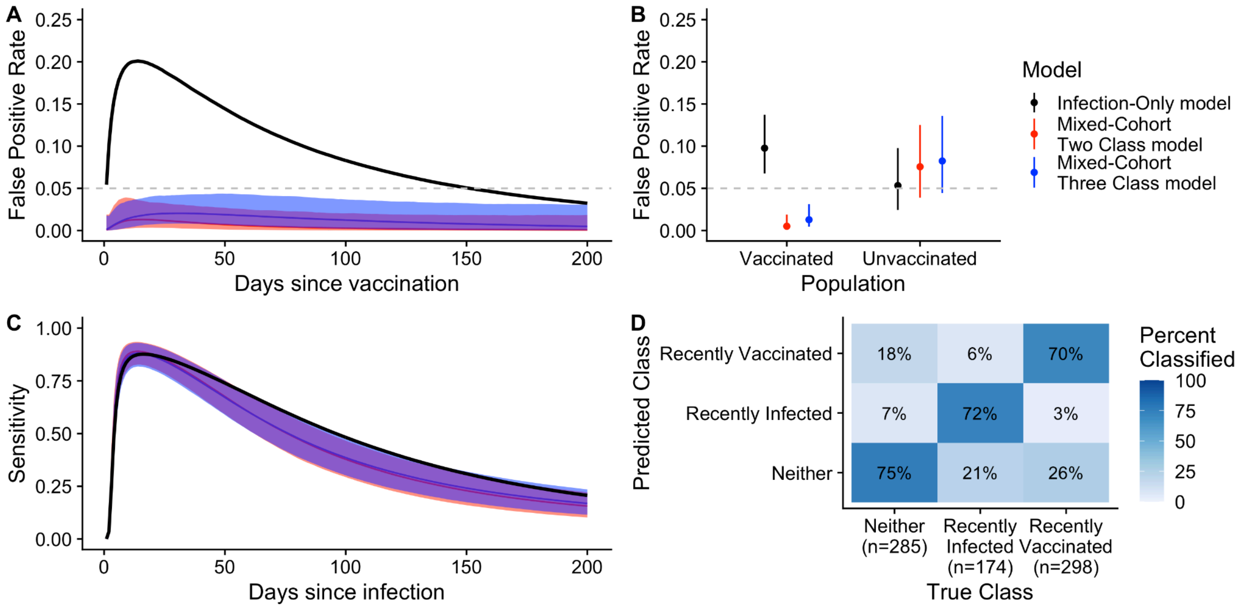

Supplement: Supplemental material — Supplemental text, tables, and figures. [file mbio.01898-25-s0001.pdf]
